# Foreign Intelligence

**Published:** 1866-04

**Authors:** 


*Foreign Intelligence.*---The School of Medicine founded by Maximilian, in Hapsburg, Mexico, has been attended by 200 students, but as seven years are required to complete their studies, it is doubtful if any of them graduate under his imperial patronage.

Prof. Romberg, of Berlin, lately received the congratulation of his colleagues through a deputation composed of Prof. Grafe, Griesinger, Langenbeck, Traube and Virchow, on the celebration of his 70th birthday.

At the suggestion of Prof. Rokitansky a committee, consisting of Profs. Wedl, Roll and Klob, has been appointed by the Imperial Society of Physicians in Viena to study and report upon Trichiniasis. Of the 350 persons affected during the late epidemic at Hedersleben, more than 90 have died. The village contained but 1800 inhabitants.---*Boston Medical and Surgical Journal.*

